# Exploring the Multifaceted Causes of Ischemic Stroke: A Narrative Review

**DOI:** 10.7759/cureus.47531

**Published:** 2023-10-23

**Authors:** Chirag Gupta, Vasant Wagh

**Affiliations:** 1 Community Medicine, Jawaharlal Nehru Medical College, Datta Meghe Institute of Higher Education and Research, Wardha, IND

**Keywords:** atherosclerosis, acute, artery, stroke, ischemia

## Abstract

Neurologists are well-versed with acute ischemic stroke, a serious public health concern. Effective acute stroke treatment is built on the rapid application of reperfusion therapy. This calls for prompt symptom recognition by the general population as well as emergency workers, proper referral to specialized stroke centers, and thorough examination and assessment by the on-site stroke team. The main goal of treatment for certain individuals is to restore blood flow to the ischemic penumbra by using intravenous thrombolysis and/or endovascular thrombectomy. Acute stroke patients must be hospitalized and continuously monitored for early neurological decline in order to avoid subsequent problems. After swiftly determining the stroke mechanism, patients can start the proper secondary preventative actions.

## Introduction and background

Acute stroke is the third most frequent cause of sickness and death worldwide, placing a significant load on the healthcare system. More than 750,000 recorded cases and over 140,000 fatalities each year are linked to stroke in the United States alone [1]. When patients enter with acute stroke symptoms in hospital emergency rooms, it is normal to rapidly do computed tomography (CT) scans, frequently before a thorough clinical evaluation (NIHSS - National Institutes of Health Strokes Scale). Rapid imaging is essential for a timely stroke diagnosis. These imaging patterns can also provide important information about the underlying causes of cerebral vascular accidents, which can be used to guide the choice of secondary preventative drugs and quick therapies for stroke recovery. For quick picture interpretation and additional imaging, proficiency in spotting these patterns is essential to make quick decisions [2]. According to a thorough meta-analysis from 2011, 11% of people had another stroke within a year after their first one, and this number rose to 26% within five years [3]. The bulk of the studies used in this analysis only tracked patients for up to one year after a stroke, and only seven of them gave information on recurrence rates after five years. The meta-analysis's authors claim that the likelihood of recurrence at five years decreased by almost half between 1961 and 2006 [4]. Recent thorough studies have shown that secondary preventative measures such as the use of statins, lifestyle modifications, and a combination of anti-platelet and anti-coagulant drugs can lower the incidence of ensuing vascular events by 20% to 30% [5].

## Review

Methodology

**Figure 1:**
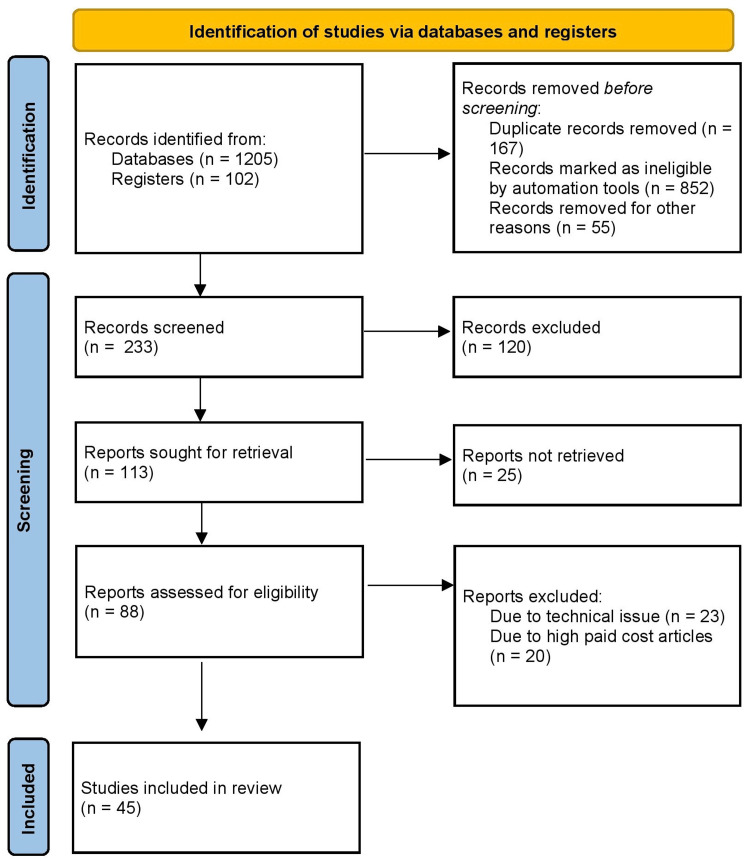
Search strategy followed for the review.

For this narrative review on ischemic stroke, we conducted a thorough search across three key databases: PubMed, Cochrane Library, and Google Scholar, covering articles available until July 2023. Our search utilized a systematic approach involving relevant keywords such as “stroke,” “ischemic stroke,” “atherosclerosis,” “hypertension,” “imaging,” and “NIHSS examination,” combined with Boolean operators. We adhered to Cureus journal criteria, focusing on peer-reviewed articles involving adult populations and examining the connection between ischemic stroke and atherosclerosis, hypertension, imaging, and clinical examination. Following the screening of titles and abstracts by us and a subsequent full-text evaluation, we included a total of 45 articles for this narrative review. Data extracted encompassed study design, sample size, demographics, findings, and relevant statistical data. Quality assessment was conducted using established tools. The findings were synthesized into key themes, encompassing the roles of atherosclerosis and hypertension in ischemic stroke, types, diagnostic imaging techniques, and clinical examination methods. Ethical approval was not required. Figure 1 shows the search strategy used in this review.

Causes of ischemic strokes 

Most infarcts are caused by ischemic strokes, which can be separated based on the reason. The Trial of Org 10172 in Acute Stroke Treatment (TOAST) method was created to classify ischemic stroke subtypes and direct optimal management [6]. 

Acute stroke instances are significantly influenced by extensive vascular disease, which is responsible for 30% to 43% of such occurrences [7]. It develops when atherosclerosis in the cervical or proximal intracranial arteries begins to form; frequently resulting in infarctions greater than 15 to 20 mm^2^. The cortex, cerebellum, brainstem, and subcortical regions are all affected by these infarctions, among other locations. Evaluating the cervical and cerebral vasculature becomes essential when there is a suspicion that significant artery atherosclerosis is the underlying cause of an acute infarction [8]. Carotid atherosclerosis-related territorial infarctions typically damage the cortex and deep grey matter. They mainly affect the area anteriorly supplied by the middle cerebral arteries (MCA). These infarcts often damage the anterior parietal lobe, posterior frontal lobe, and superior temporal lobe. It is crucial to recollect that proximal cerebral vascular disease is also included in big artery atherosclerosis [9].

Large Artery Atherosclerosis

Stroke is thought to be caused by a number of different processes, including artery-to-artery emboli, in situ thromboembolism, and hemodynamic dysfunction, especially in people with severe vascular atherosclerosis. When this intricate pathophysiology affects the vertebrobasilar system, unique imaging patterns result. Bilateral infarctions are occasionally seen in the midbrain, and they are typically accompanied by severe arterial atherosclerosis that causes distal basilar occlusions, especially affecting the midbrain and thalami [10]. Specialists must carefully examine the distal vertebral artery because obstruction of the posterior inferior cerebellar artery frequently causes lateral medullary infarction, which presents clinically as lateral medullary syndrome. These infarcts frequently have hemodynamic problems and proximal stenosis in common as the major cause of CVA. The intersection of the anterior, middle, and posterior cerebral arteries (PCAs) is prone to the development of cortical lesions known as external border-zone infarcts. Localized decreased blood flow or hemodynamic impairment brought on by stenosis in the midbrain may have an impact on these infarcts. Embolic occurrences might not be effectively cleansed in such circumstances. The lowest perfusion pressure, however, is found in internal border-zone infarcts that are supplied by the lenticulostriate arteries [11]. When there is a fetal PCA, posterior circulation border zone infarcts are less common, and when they do occur, an embolic source from the anterior circulation is often thought to be the main cause [12].

Cardioembolic

20% to 31% of acute ischemic infarctions are caused by cardioembolic factors. These infarcts frequently appear on magnetic resonance imaging (MRI) as several bilateral foci with constrained diffusion, affecting different vascular regions [13]. It is vital to take further cardiac examination, including extra diagnostic imaging, into account when this imaging pattern is seen. Cardioembolic episodes can be caused by a number of diseases, including atrial fibrillation, myocardial infarction with left ventricular thrombus, patent foramen ovale, atrial septal aneurysm, and inflammatory or infectious endocarditis. In addition, emboli and bilateral infarctions can also be caused by aortic arch diseases, which are commonly missed during standard transthoracic echocardiography [14].

Even if the precise origin of the most prevalent etiology may be up for discussion, it is crucial to consider a possible cardioembolic source when isolated anterior cerebral artery (ACA) infarctions are discovered. While some research implies that isolated ACA infarctions and cardioembolism are more frequently linked, other studies show that ACA's major artery atherosclerosis is a more prevalent cause. It is important to remember that isolated ACA infarctions are less frequent than MCA infarctions [10]. Both ACA and MCA areas are commonly affected by cardioembolic disorders, which are the source of infarctions. For instance, distal emboli may form in an atrial fibrillation patient who suffers from an acute myocardial infarction and may suffer a reduced cardiac output affecting ACA and MCA regions [15].

Small Artery Occlusion

Small artery occlusions are a potential cause in situations when infarcts are less than 20 mm, and there is no indication of other illnesses such as vasospasm or a cardioembolic source. Acute strokes are caused by these occlusions in 10% to 23% of instances. Smoking, sedentary lifestyle, and age are risk factors for minor artery occlusions. Most frequently, lacunar infarcts are seen in the basal ganglia, internal capsule, corona radiata, and brainstem. Infarcts in the PCA area frequently have lacunar origins and more significant clinical manifestations. To diagnose minor artery occlusions correctly, proximal vascular artery inclusion must be ruled out [16]. Secondary imaging anomalies may offer more proof that the infarction was brought on by small artery disease. These abnormalities may include cerebral atrophy, substantial perivascular spaces, white matter ischemic illness, cerebral hemorrhage, chronic lacunar infarcts, and chronic lacunar infarcts [17].

Vasculopathies

Territorial infarctions affecting the basal ganglia and deep white matter fed by perforating vessels frequently coincide with diseases affecting the proximal arteries. Moyamoya disease is an example of a notable disease with extensive collateral circulation. Both children and adults can get the Moyamoya disease, which causes frequent and significant territorial infarctions. Children are more likely to develop ischemic infarctions than adults, who usually suffer from hemorrhagic infarctions. Similar to tuberculosis (TB), basilar meningitis can cause proximal vasospasms, which may cause CVA [18]. Basilar meningitis differs from Moyamoya in that it can cause infarctions in the thalamus and basal ganglia as a result of perforating artery involvement, with more evidence supporting this origin [19]. Infarctions have been related to acute septic meningitis brought on by Streptococcus pneumoniae, Neisseria meningitidis, Haemophilus influenzae, and Staphylococcus aureus, and vascular imaging may occasionally show vasospasm. When second and third-order arteries are affected, it is possible for cortical infarctions to occur in distant arterial regions, frequently with the possibility of bleeding. Potential causes of arterial wall enhancement seen on contrast-enhanced MR angiography include sarcoidosis, polyarteritis nodosa, primary angiitis of the CNS (PACNS), and granulomatosis with polyangiitis [20]. Additionally, second and third-order arteries can be affected by viral diseases like syphilis and herpes zoster, and imaging outcomes may match those seen in PACNS during herpes zoster reactivation. Initial symptoms frequently involve a number of infarctions, such as those in the basal ganglia and subcortical white matter. The proximal MCA and anterior ACA may exhibit segmental stenosis, thrombosis, or beading during angiography [21].

Angiography frequently shows no abnormalities in small vessel vasculopathies such as Sjogren's syndrome, collagen vascular disease, and human immunodeficiency virus (HIV) encephalitis. Deep grey structures, white matter, and subcortical areas are susceptible to infarctions. White matter lesions in small vessels typically line up parallel to the ventricles, setting them apart from multiple sclerosis. These lesions frequently appear early in life and advance quickly. Systemic lupus erythematosus (SLE), a collagen vascular disease, can potentially be a complication that causes small vessel vasculopathy. The infarction pattern in lupus is non-specific. Secondary causes of the illness, such as heart valve disease (Libman-Sacks endocarditis), hypercoagulability, atherosclerosis brought on by high blood pressure and steroid therapy, and venous thrombosis, are more frequently linked to infarctions than vasculitis [22].

Acute infarcts may form in individuals with neoplasms due to vascular invasion by the tumor. Vascular blockage may be found during an angiogram. Diffuse large B-cell lymphoma (DLBCL), a kind of non-Hodgkin lymphoma, is one of the most prevalent causes of neoplastic vasculopathy. Acute infarcts and infarct-like lesions demonstrating diffusion limitation on MR imaging and signal anomalies on T2-weighted sequences can occur in patients with DLBCL. The corpus callosum and periventricular white matter may be impacted by infarcts, which might be dispersed in a watershed pattern. Since DLBCL often affects tiny arteries, angiography is frequently done to confirm the diagnosis. Meningeal enhancement and central pontine hyperintensity are examples of secondary results that, in some circumstances, might be attributable to venous circulation [23]. 

Acute infarction can also be caused by inherited factors. The anterior temporal lobes, superior frontal lobes, and external capsule are the primary sites of localized and subcortical white matter infarcts in cerebral autosomal dominant arteriopathy with subcortical infarcts and leukoencephalopathy [24].

Dissection

Unexpected cases of stroke can be linked to arterial dissection, a relatively rare but important illness. Younger people are typically affected by this condition, which frequently manifests as headaches, neck pain, and trouble concentrating. The main factor that frequently causes arterial dissection is trauma. Emboli are more likely than hypoperfusion to result in cerebral infarction in cases of arterial dissection. Vascular imaging must be used to diagnose arterial dissection since it can show whether a dissection flap is present and whether stenosis or occlusion is the result of the dissection. A thorough assessment of the condition requires thorough vascular imaging. Notably, a distal cervical internal carotid artery (ICA) dissection should be properly studied and may be visible with conventional brain MRI [25].

Embolic Stroke of Undetermined Source

This group is referred to when several causes or no causes of stroke are identified. It is essential to consider potential causes of cryptogenic stroke as those that routine cardiac imaging, cardiac workup, or vascular imaging may miss. These disorders include aortic arch atherosclerosis, paroxysmal atrial fibrillation, non-stenosis atherosclerosis of the carotid arteries, and patent foramen ovale [26].

Unusual Causes

2% to 11% of ischemic strokes have unusual causes [27]. Even though they are uncommon, it is crucial to investigate unusual reasons once the usual ones have been ruled out since they may have substantial treatment consequences. Vasculopathies, along with other factors such as hypercoagulable illnesses, blood disorders, right-to-left vascular shunts, and arterial dissections, make up a sizeable fraction of the less common causes of stroke. Even though many of these medical diseases have visual traits, specific vascular patterns, and additional imaging results can reveal the true cause of the issue [28]. 

Diagnosis

Initial History

In order to make a diagnosis, history is crucial in any profession, including neurology. However, information must be acquired promptly in the event of an acute stroke, emphasizing resolving a small number of crucial concerns. Due to the nature of their disability, patients usually find it challenging to give a trustworthy history; thus, supporting evidence from family members and nearby witnesses should be taken. Patients' last health check-up also has to be documented [29]. 

Early verification of whether the patient is inside the reperfusion therapy treatment window will affect the scheduling of upcoming investigations and the prioritization of concurrent referrals. The moment the symptoms initially manifested should be noted to avoid misdiagnosis. Unseen occurrences or 'wake-up' strokes should be documented at the exact time the patient was last healthy (as opposed to when they were discovered), using an action as a stand-in, such as getting up to use the phone or the lavatory, might be beneficial. The timing of the early symptoms also has to be documented. However, there are notable exceptions, such as the stammering quality of the capsular warning syndrome or the foregoing symptoms of basilar artery blockage. Stroke symptoms often develop unexpectedly. In the first few hours after a stroke, changing severity is typical, and early improvement may be followed by worsening, particularly in patients with intracranial artery occlusion. The more gradual onset of the symptoms might point to an alternative diagnosis. Significant drug or medication histories and allergies have to be documented, too [30]. A quick assessment of the patient's history, especially vascular risk factors, will impact the diagnosis; in certain situations, this information may be obtained through electronic medical records before the patient's arrival. Risk factors for ischemic stroke include cigarette smoking, high blood pressure, high cholesterol, diabetes mellitus, peripheral vascular disease, and drug abuse [31]. 

A history of atrial fibrillation or carotid stenosis can offer crucial hints about the underlying cause of a stroke. By analyzing the patient's medication list, it is possible to assess whether any relevant medical problems, stroke risk factors, and whether the patient is currently receiving oral anticoagulation therapy will have an impact on the decision to administer thrombolysis. It is important to remember that 20% to 25% of first presentations that were initially misdiagnosed as strokes actually turned out to be illnesses that mirror strokes, many of which can be recognized through the patient's medical history. In one study, the five most frequent stroke mimics were seizures, syncope, sepsis, migraines, and brain tumors [32]. The symptoms of posterior circulation strokes are frequently hazy, such as sporadic headaches or vertigo (disequilibrium). They are misdiagnosed three times as often as anterior circulation strokes. Further testing should be done if a patient exhibits vertigo or disorientation coupled with other symptoms connected to the posterior circulation and an abrupt start.

Examination and Findings

A headache or isolated “dizziness” (vertigo or disequilibrium) are two common symptoms of posterior circulation strokes. Further testing should be done when a patient exhibits vertigo or disorientation coupled with other symptoms connected to the posterior circulation and an abrupt start [33]. Fever may be a sign of a number of illnesses, including infective endocarditis, aspiration pneumonia, or a urinary tract infection. Instead of performing a thorough examination, it is crucial to concentrate on pinpointing the vascular region that is impacted and measuring physical damage during the NIHSS test [34]. Up to 80% of patients experience an acute ischemic stroke, and elevated blood pressure (>140 mmHg systolic), even though it may gradually normalize over time, is linked to poorer outcomes like intracerebral hemorrhage in the following week [35]. Unknown factors that could contribute to temporary post-stroke hypertension include altered cerebral autoregulation and non-stroke causes like urine retention and psychological stress. While lacunar stroke syndromes are typically linked to cerebral small-vessel illness, large-vessel stroke syndromes suggest an atheroembolic etiology. The clumsy hand-dysarthria syndrome, which can also be cortical, ataxic hemiparesis, contralateral pure motor, pure sensory, and sensorimotor dysfunction, are all lacunar syndromes [36].

The head impulse nystagmus test of skew (HINTS) bedside examination is frequently used to assess individuals presenting with acute vestibular disorders. It is a three-step process that has been effective in determining the underlying cause, especially when separating central from peripheral etiologies. The HINTS test exhibits a high sensitivity of 100% and specificity of 96% in finding a core cause [37]. Due to the solitary aberrant head impulse test's less positive predictive value of 69% (which shows unilateral peripheral vestibulopathy), it is crucial to manage it cautiously. Even though a positive head impulse test is more suggestive of a peripheral origin, more testing is required to rule out probable central causes and provide a firm diagnosis [38].

Imaging

Stroke centers should adopt guidelines to reduce neuroimaging delays and guarantee prompt diagnosis and care. Two practices that can be useful in accomplishing this aim are the use of protocolized stroke imaging sequences and prioritizing the use of a dedicated scanner close to the emergency room. The primary modality for acute stroke in the hyperacute scenario is CT-based neuroimaging. Making judgments on thrombolysis usually only requires a non-contrast CT scan of the head since it can quickly, accurately, and affordably rule out cerebral hemorrhage. It is crucial to remember that due to the fluctuation in tissue water content and the apparent change in parenchymal attenuation during the hours after ischemia, CT scanning has limits in terms of its sensitivity and specificity for diagnosing acute ischemia [39]. While MRI and other imaging modalities can offer more accurate information regarding the degree and location of ischemia damage, CT imaging is essential for early evaluations. To reduce wait times and improve stroke diagnosis and treatment precision, stroke treatment facilities should work to make the most of neuroimaging techniques. It is significant to highlight that the specificity of CT imaging might be hampered by pre-existing ischemia changes or persistent infarcts. The loss of the grey-white matter distinction, hemispheric sulcal effacement, loss of the integrity of the lentiform nucleus, or hyperdensity inside an intracranial artery are only a few signs of ischemia that can be seen on a non-contrast CT scan (dense artery sign) [40]. Alberta Stroke Program Early CT score (ASPECTS), a 10-point scale developed by the Alberta Stroke Program, can measure early ischemia alterations and determine the degree of parenchymal damage. Stroke centers can improve their capacity to precisely identify and characterize ischemic lesions by utilizing a variety of imaging modalities [41]. To enable practical interpretation of imaging data and save needless delays while giving rtPA (alteplase), stroke centers must have established protocols in place when endovascular thrombectomy is a possible course of therapy, cervicocranial and intracranial artery CT-angiography should be done right once to check for intracranial artery occlusion. Although current randomized research is being undertaken to investigate this better, observational evidence shows that individuals with non-disabling symptoms brought on by cerebral major artery blockage may benefit from thrombolysis. A poor diagnosis is linked to minor strokes and Transient Ischemic Attacks (TIAs), including intracranial major artery blockage. In stroke centers, having well-established protocols for interpreting imaging results and prompt delivery of necessary medications is essential to maximizing patient outcomes [42].

To assess various facets of cerebral perfusion during CT-perfusion sequences, automated software can be used, such as MIStar from Apollo Medical Imaging Technology or Rapid Processing of Perfusion and Diffusion (RAPID) CT-perfusion offered by iSchemaView. CT perfusion, commonly referred to as cerebral perfusion CT (CPT), is used to differentiate between penumbra and ischemia and assessment of collateral blood flow [43].

MRI has a substantially higher sensitivity for detecting ischemia as compared to CT scans, especially in situations of mild strokes. This increased sensitivity is crucial in making accurate predictions of adverse short- and long-term outcomes. In MRI techniques, a variety of sequences are frequently used to thoroughly analyze the brain. These procedures include diffusion-weighted imaging (DWI), rapid stroke T2-fluid-attenuated inversion recovery (FLAIR), time-of-flight MR-angiography for intracranial arteries, and blood-sensitive procedures like gradient-recalled echo or susceptibility-weighted imaging [44]. Healthcare providers can learn crucial details regarding cerebral perfusion, ischemic lesions, and the length of symptom onset by integrating these cutting-edge imaging technologies and implementing the relevant sequences into MRI procedures [45].

## Conclusions

Given that acute ischemic stroke is a severe public health issue, aspiring neurologists must have a thorough understanding of the condition. In order to properly send patients to specialist stroke centers, it is crucial for the general public and emergency workers to recognize stroke symptoms as soon as possible. For best management, swift and efficient examination by specialist stroke teams is essential. The underlying etiology of cerebral ischemia, which informs the choice of preventative medications and therapeutic approaches for stroke recovery, can be identified by imaging patterns. It is important to remember that many studies have scant information on recurrence after five years and scant follow-up data beyond one year. However, recent initiatives have produced encouraging outcomes in lowering the risk of stroke recurrence over time. The most frequent form of strokes is ischemic strokes, which can be categorized according to their underlying causes. A significant number of cases are caused by sizeable vascular diseases primarily caused by atherosclerosis in the cervical or cerebral arteries. When there is a suspicion of atherosclerosis in a major artery, the cervical and cerebral vasculature has to be adequately evaluated. Future neurologists may improve patient outcomes and successfully address this significant public health issue by remaining current with recent developments and understanding acute ischemic stroke. At the confluence of cerebral arteries, border zone infarcts develop and are probably brought on by a combination of embolic incidents and hypoperfusion. A major part of acute ischemic infarctions is caused by cardioembolic factors, with significant artery atherosclerosis also contributing. Small artery disease is indicated by lacunar infarcts, which preferentially damage particular brain areas. Vascular diseases can cause territorial infarcts, although sickle cell disease and moyamoya have collateral circulation. Vascular risk factors and symptoms must be evaluated quickly and precisely for a precise diagnosis. Stroke centers should avoid neuroimaging delays, and CT imaging is essential in diagnosing cases of ischemic strokes more efficiently.
